# Amniotic Fluid and Amniotic Membrane Stem Cells: Marker Discovery

**DOI:** 10.1155/2012/107836

**Published:** 2012-06-03

**Authors:** Maria G. Roubelakis, Ourania Trohatou, Nicholas P. Anagnou

**Affiliations:** ^1^Laboratory of Biology, University of Athens School of Medicine, 115 27 Athens, Greece; ^2^Cell and Gene Therapy Laboratory, Centre of Basic Research II, Biomedical Research Foundation of the Academy of Athens (BRFAA), 115 27 Athens, Greece

## Abstract

Amniotic fluid (AF) and amniotic membrane (AM) have been recently characterized as promising sources of stem or progenitor cells. Both not only contain subpopulations with stem cell characteristics resembling to adult stem cells, such as mesenchymal stem cells, but also exhibit some embryonic stem cell properties like (i) expression of pluripotency markers, (ii) high expansion in vitro, or (iii) multilineage differentiation capacity. Recent efforts have been focused on the isolation and the detailed characterization of these stem cell types. However, variations in their phenotype, their heterogeneity described by different groups, and the absence of a single marker expressed only in these cells may prevent the isolation of a pure homogeneous stem cell population from these sources and their potential use of these cells in therapeutic applications. In this paper, we aim to summarize the recent progress in marker discovery for stem cells derived from fetal sources such as AF and AM, using novel methodologies based on transcriptomics, proteomics, or secretome analyses.

## 1. Introduction

Both amniotic fluid (AF) and amniotic membrane (AM) represent rich sources of stem cells that can be used in the future for clinical therapeutic applications. Ethical concerns regarding the isolation of stem cells from these sources are minimized [1–3], in contrary to the issues emerging from human embryonic stem cell (ESC) research [4–6]. AF is collected during scheduled amniocenteses between 15th and 19th week of gestation for prenatal diagnosis and the excess of sample can be used for cell sourcing [2, 4–9], whereas AM is usually collected during the caesarean sections of term pregnancies [10, 11]. Given the heterogeneity of the stem cell populations derived from these sources, the isolation of specific cell types is difficult and requires a detailed phenotypic and molecular characterization of the respective cells. Studies that include *omics* approaches are fundamental in better understanding the mechanisms of molecular expression of these cells and defining the correct methodologies for their isolation, prior to their use in therapeutic approaches.

This paper aims to present the main biological and molecular characteristics of AF- and AM-derived stem cells and also to highlight the recent advances in marker discovery using global methodologies, such as transcriptomics, proteomics, or secretome analyses.

### 1.1. Amniotic Fluid

AF serves as a protective liquid for the developing embryo, providing mechanical support and the required nutrients during embryogenesis [1, 3]. Amniocentesis has been used for many decades as a routine procedure for fetal karyotyping and prenatal diagnosis, allowing the detection of a variety of genetic diseases [1, 3, 12].

The major component of AF is water; however its overall composition varies throughout pregnancy. At the beginning of pregnancy, the amniotic osmolarity is similar to the fetal plasma. After keratinization of the fetal skin amniotic osmolarity decreases relatively to maternal or fetal plasma, mainly due to the inflow of fetal urine [1]. More interestingly, AF also represents a rich source of a stem cell population deriving from either the fetus or the surrounding amniotic membrane [1, 12]. Additional investigations by several groups have been recently focused on the cellular properties of amniotic derived cells and their potential use in preclinical models [13–18] and in transplantation therapies [7, 17, 19–24].

#### 1.1.1. Amniotic Fluid Stem Cells (AFSCs)

The amniotic fluid cells (AFCs) represent a heterogeneous population derived from the three germ layers. These cells share an epithelial origin and are derived from either the developing embryo or the inner surface of the amniotic membrane, which are characterized as amniotic membrane stem cells [12]. The AFCs are mainly composed of three groups of adherent cells, categorized based on their morphological, growth, and biochemical characteristics [12]. Epithelioid (E-type) cell are cuboidal to columnar cells derived from the fetal skin and urine, amniotic fluid (AF-type) cells are originating from fetal membranes, and fibroblastic (F-type) cells are generated mainly from fibrous connective tissue. Both AF- and F-type cells share a fibroblastoid morphology and the dominant cell type appears to be the AF-type, coexpressing keratins and vimentins [1–3, 8, 9, 25–27]. Several studies have documented that human amniotic fluid stem cells (AFSCs) can be easily obtained from a small amount of second trimester AF, collected during routine amniocenteses [2, 4–9], a procedure with spontaneous abortion rate ranging from 0.06 to 0.5% [2, 28, 29]. Up to date, a number of different cultivation protocols have been reported, leading to enriched stem cell populations. The isolation of AFSC and the respective culture protocols were summarized in a recent review by Klemmt et al. [3] and can be categorized as follows: (i) a single step cultivation protocol, where the primary culture was left undisturbed for 7 days or more until the first colonies appear [2, 3, 30–32], (ii) a two-step cultivation protocol, where amniocytes, not attached after 5 days in culture, were collected and further expanded [3, 5, 33], (iii) cell surface marker selection for CD117 (c-kit receptor) [3, 7, 34, 35], (iv) mechanical isolation of the initial mesenchymal progenitor cell colonies formed in the initial cultures [9], and (v) short-term cultures to isolate fibroblastoid colonies [36]. The majority of the AFSCs, isolated following these methodologies, shared a multipotent mesenchymal phenotype and exhibited higher proliferation potential and a wider differentiation potential compared to adult MSCs [2, 4–7, 9, 24, 37].

### 1.2. Amniotic Membrane (AM)

The amniotic membrane, lacking any vascular tissue, forms most of the inner layer of the fetal membrane [12, 38] and is composed of 3 layers: (i) an epithelial monolayer consisting of epithelial cells, (ii) an acellular intermediate basement layer, and (iii) an outer mesenchymal cell layer, rich in mesenchymal stem cells and placed in close proximity to the chorion [12, 38]. AM was used in clinic for many decades for wound healing in burns, promoting epithelium formation and protecting against infection [39, 40]. Recently, the use of AM has been evaluated as a wound dressing material for surgical defects of the oral mucosa [41], ocular surface reconstruction [40, 42], corneal perforations [43, 44], and bladder augmentation [45].

#### 1.2.1. Amniotic Membrane Stem Cells (AMSCs)

Amniotic membrane stem cells (AMSCs) include two types, the amniotic epithelial cells (AECs) and the amniotic membrane mesenchymal stem cells (AM-MSCs) derived from the amniotic epithelial and the amniotic mesenchymal layers, respectively [12, 46]. Both cell types are originated during the pregastrulation stages of the developing embryo, before the delineation of the three primary germ layers and are mostly of epithelial nature [38, 47]. A variety of protocols have been established for AECs and AM-MSCs isolation, primarily based on the mechanical separation of the AM from the chorionic membrane and the subsequent enzymatic digestion [47–50]. AM-MSCs exhibited plastic adherence and fibroblastoid morphology, while AECs displayed a cobblestone epithelial phenotype. AM-MSCs shared similar phenotypic characteristics with the ones derived from adult sources. More interestingly, AM-MSCs, similarly to AF-MSCs, exhibited a higher proliferation rate compared to MSCs derived from adult sources [12, 51] and a multilineage differentiation potential into cells derived from the three germ layers [27].

## 2. Immunophenotype

### 2.1. Amniotic Fluid Stem Cells

The AF has recently emerged as an alternative fetal source of a variety of cells of stem cell origin [1, 3]. Herein, we aim to summarize the key markers that characterize AFSCs. To date, MSCs represent the best characterized subpopulation of AFSCs. The AF-MSCs exhibited typical mesenchymal marker expression, such as CD90, CD73, CD105, CD29, CD166, CD49e, CD58, and CD44, determined by flow cytometry analyses [2, 5–8, 10, 12, 21, 32, 33, 52, 53]. Additionally, these cells expressed the HLA-ABC antigens, whereas the expression of the hematopoietic markers CD34 and CD45, the endothelial marker CD31, and the HLA-DR antigen was undetected [2, 5, 6, 32]. More importantly, the majority of cultured AF-MSCs expressed pluripotency markers such as the octamer binding protein 3/4 (Oct-3/4), the homebox transcription factor Nanog (Nanog), and the stage-specific embryonic antigen 4 (SSEA-4) [2, 5–7, 9, 21, 32, 33, 52].

It was also reported that amniocyte cultures contain a small population of CD117 (a tyrosine kinase specific for stem cell factor present primarily in ESCs and primordial germ cells) positive cells that can be clonally expanded in culture [7]. The differentiation properties of CD117^+^ AFS were tested for the first time in vivo, proving in this way their stem cell identity [7]. Experimental evidence suggested that AFSCs are derived from spindle-shaped fibroblastoid cells [10].

In an attempt to analyze the AFSCs subpopulations, our group recently identified two morphologically distinct populations of AFSCs of mesenchymal origin, with different proliferation and differentiation properties, termed as spindle shaped (SS) and round shaped (RS) [9]. Both subpopulations were expressing mesenchymal stem cell markers at similar levels. However, it was identified that SS colonies expressed higher levels of CD90 and CD44 antigens compared to RS colonies [9].

### 2.2. Amniotic Membrane Stem Cells (AMSCs)

A detailed immunophenotype analysis of AMSCs revealed the expression of antigens, such as CD13, CD29, CD44, CD49e, CD54, CD73, CD90, CD105, CD117^low^, CD166, CD27^low^, stromal stem cell marker 1 (Stro-1), SSEA-3, SSEA-4, collagen I and III (Col1/Col3), alpha-smooth muscle actin (*α*-SMA), CD44, vimentin (Vim), fibroblast surface protein (FSP), and HLA-ABC antigen [10, 12, 27]. However, intercellular adhesion molecule 1 (ICAM-1) was expressed in very low levels and proteins TRA-1-60, vascular cell adhesion protein 1 (VCAM-1), von Willebrand factor (vWF), platelet endothelial cell adhesion molecule (PECAM-1), CD3, and HLA-DR were not detected [10, 27]. One of the most abundant proteins found in AM derived cells is laminin, which plays a key role in differentiation, cell shape and migration, and tissue regeneration [54, 55]. RT-PCR analysis further showed that AMSCs expressed genes, such as Oct-3/4, zinc finger protein 42 (zfp42 or Rex-1), stem cell factor protein (SCF), neural cell adhesion molecule (NCAM), nestin (NES), bone morphogenetic protein 4 (BMP-4), GATA binding protein 4 (GATA-4), and hepatocyte nuclear factor 4*α* (HNF-4*α*) even in high passages. Brachyury, fibroblast growth factor 5 (FGF5), paired box protein (Pax-6), and bone morphogenetic protein 2 (BMP2) transcripts were not detected [10, 12]. Similarly, AECs were positive for CD10, CD13, CD29, CD44, CD49e, CD73, CD90, CD105, CD117, CD166, Stro-1, HLA-ABC, and HLA-DQ^low^ and negative for CD14, CD34, CD45, CD49d, and HLA-DR expressions, as determined by FACS analyses [27, 47–50]. Further investigation showed that AECs were expressing stem cell markers such as SSEA-1, SSEA-3, SSEA-4, Nanog, sex determining region Y-box 2 (Sox2), Tra1-60 and Tra1-80, fibroblast growth factor 4 (FGF4), Rex-1, cryptic protein (CFC-1), and prominin 1 (PROM-1) [38, 50].

## 3. Transcriptomics

### 3.1. Amniotic Fluid Stem Cells

 A functional analysis of the gene expression signature of AF-MSCs compared to bone-marrow- (BM-), cord-blood- (CB-), and AM-MSCs was initially performed by Tsai et al. [11]. Genes expressed in MSCs from all three sources could be categorized in groups related to (i) extracellular matrix remodeling (CD44, collagen II (COL2), insulin-like growth factor 2 (IGF2), and tissue inhibitor of metalloproteinase 1 (TIMP1)), (ii) cytoskeletal regulation (urokinase-type plasminogen activator (PLAU) and receptor (PLAUR)), (iii) chemokine regulation and adhesion (alpha actinin 1 (ACTN1), actin-related protein complex subunit 1B (ARPC1B) and thrombospondin 1 (THBS1)), (iv) plasmin activation (tissue factor pathway inhibitor 2 (TFPI2)), (v) transforming growth factor *β* (TGF*β*) receptor signaling (caveolin 1 (Cav1), caveolin 2 (Cav2), cyclin-dependent kinase inhibitor 1A (CDKN1A)), and (vi) genes encoding E3 ubiquitin ligases (SMURF) [11]. The upregulated genes in AF-MSCs compared to BM-, CB-, and AM-MSCs included molecules involved in uterine maturation and contraction, such as oxytocin receptor (OXTR) and regulation of prostaglandin synthesis, such as phospholipase A2 (PLA2G10). Other upregulated genes in this group were involved in signal transduction related to (i) thrombin triggered response ((F2R and F2RL)), (ii) hedgehog signaling ((hedgehog acyltransferase (HHAT)), and (iii) G-protein-related pathways (rho-related GTP-binding protein (RHOF), regulator of G protein signaling 5 and 7 (RGS5, RGS7), and phospholipase C beta 4 (PLCB4)) [11].

 In recent studies on AFSCs, Kim et al. described for the first time the gene expression changes in total AFSC population during different passages by illumina microarray analysis. 1970 differentially expressed genes were detected and categorized according to their expression profiles into 9 distinct clusters [56]. Genes with gradually increasing expression levels included chemokine (C-X-C motif) ligand 12 (CXCL12), cadherin 6 (CDH6), and folate receptor 3 (FOLR3). Downregulated genes were among others, cyclin D2 (CCND2), keratin 8 (K8), IGF2, natriuretic peptide precursor (BNP) B, and cellular retinoic acid binding protein 2 (CRABPII) [56]. To obtain further information, chip data analysis on aging genes was performed and revealed upregulation of gene transcripts, such as nerve growth factor beta (NGF*β*), insulin receptor substrate 2 (IRS-2), insulin-like growth factor binding protein 3 (IGFBP-3), and apolipoprotein E (APOE). Expression of genes, such as PLAU, E2F transcription factor 1 (E2F1), IGF2, breast cancer type 1 susceptibility gene (BRCA1), DNA topoisomerase 2-alpha (TOP2A), proliferating cell nuclear antigen (PCNA), forkhead box M1 (FOXM1), cyclin-A2 gene (CCNA2), budding uninhibited by benzimidazoles 1 homolog beta (BUB1B), and cyclin dependent kinase 1 (CDC2), was gradually downregulated during culture [56].

Wolfrum et al. performed a global gene expression analysis of AFSCs compared to iPSCs derived from AF (AFiPSC) and ESCs [57]. Among these, genes related to self renewal and pluripotency (1299 genes e.g., POU class 5 homeobox 1 (POU5F1), Sox2, Nanog, microRNA-binding protein LIN28) and AFSCs-specificity (665 genes, e.g., OXTR, HHAT, RGS5, neurofibromatosis type 2 (NF2), protectin (CD59), tumor necrosis factor superfamily member 10 (TNFSF10), 5′-nucleotidase (NT5E)) were detected in AFSCs [57]. Furthermore, the authors examined the expression of senescence and telomere associated genes in AFSCs of early and later passage, in order to study the effect of reprogramming on bypassing senescence observed in AFSC cultures. Sixty-four genes were identified as differentially expressed in AFSCs compared to AFiPSC lines. Of these, telomere-associated genes and genes involved in regulating cell cycle, such as the mitotic arrest deficient-like 2 (MAD2L2), the poly ADP-ribose polymerase 1 (PARP1), replication protein A3 (RPA3), the dyskeratosis congenita 1 (DKC1), the mutS homolog 6 (MSH6), the CHK1 checkpoint homolog (CHEK1), the polo-like kinase 1 (PLK1), the POU class 2 homeobox 1 (POU2F1), the CDC2, the Bloom syndrome gene RecQ helicase-like (BLM), the Werner syndrome RecQ helicase-like (WRN), the DNA methyltransferase 1 (DNMT1), the DNA methyltransferase 3 beta (DNMT3B), the lamin B1 (LMNB1), and the DNA replication factor 1 (CDT1), were downregulated in AFSCs compared to AFiPSCs and ESCs. In contrast, peptidylprolyl cis/trans isomerase (PIN1), lamin A/C (LMNA), growth arrest and DNA damage inducible alpha (GADD45A), chromobox homolog 6 (CBX6), NADPH oxidase 4 (NOX4), endoglin (ENG), histone H2B type 2-E (HIST2H2BE), CDKN1A, CDKN2A growth differentiation factor 15 (GDF15), and serine protease inhibitor 1 (SERPINE1), among others, were upregulated in AFSCs compared to AFiPSCs and ESCs [57].

### 3.2. Amniotic Membrane Stem Cells

Transcriptomic analysis using DNA microarrays has been reported for AM-MSCs [11]. These experimental data provided information on the AM-MSC gene expression pattern compared to gene expression profiles of AF, CB, and BM-MSCs. Several upregulated genes in AM-MSCs involved in immune adaptation regulation between the maternoplacental interface were identified. Among others, spondin 2 (SPON2), interferon, alpha inducible protein 27 (IFI27), bradykinin receptor B1 (BDKRB1), small inducible cytokine subfamily B member 5 and 6 (SCYB5, SCYB6), and Yamaguchi sarcoma viral-related oncogene homolog (LYN) were found to be upregulated [11]. In addition, other genes with increased expression in AM-MSCs compared to AF, CB, and BM-MSCs included (i) transcription factors, such as forkhead box F1 (FOXF1), heart and neural crest derivatives expressed 2 (HAND2), and transcription factor 21 (TCF21) and (ii) metabolic enzymes, such as dipeptidyl-peptidase 6 (DPP6), tryptophan 2,3-dioxygenase (TDO2), and sialyltransferases (STs) [11].

## 4. Proteomics

### 4.1. Amniotic Fluid Stem Cells

Proteomic studies on the total AFSC population, including epithelioid (E-type), amniotic fluid specific (AF-type), and fibroblastic (F-type) cells, revealed 2400 spots that resulted in the identification of 432 different gene products. The majority of the proteins was localized in cytoplasm (33%), mitochondria (16%), and nucleus (15%) and represented mainly enzymes (174 proteins) and structural proteins (75 proteins). A relatively high percentage of membrane and membrane-associated proteins were also present (7%) [58]. Among the detected proteins, 9 were corresponding to epithelial cells, such as ATP synthase D chain (ATP5H), NADH-ubiquinone oxidoreductase 30 kDa subunit (NUIM), annexin II (Anx2), annexin IV (Anx4), 40S ribosomal protein SA (Rpsa), glutathione S-transferase P (GSTP), major vault protein, and cytokeratins 19 and 7 (CK-19, CK-7), whereas 12 proteins were reported to be expressed in fibroblasts, including fibronectins, tropomyosins, transgelin (TAGLN), arp2/3 complex 34 kDa subunit (P34-arp), gelsolin (Gsn), elongation factor 1-*β* (EF-1*β*), and others. Eight proteins were found to be expressed in keratinocytes, including keratins, ribonucleoproteins, Anx2, aetyl-CoA acetyl-transferase (ACAT1), and others, three to be expressed in epidermis, including tropomyosins and keratins and one in mesenchymal cell type (vimentin 1 (Vim 1)) [58].

Recent studies provided evidence that a diversity of metabolic enzyme expression in the amnion cells is involved in metabolic and genetic syndromes, and thus, their detection might be important for prenatal diagnosis. A more detailed analysis for determining specific metabolic enzymes present in AFSCs was reported by Oh et al. [59]. Ninety-nine proteins had been identified, such as carbohydrate handing enzymes, amino acid handling enzymes, proteins of purine metabolism, and enzymes of intermediary metabolism [59, 60].

A proteomic analysis was also performed on different culture passages of CD117^+^ AFSCs, exhibiting variations in protein expression that mainly occurred in early passages [35]. Twenty-three proteins were differentially expressed between early and late passages with the most sticking downregulated proteins, the Col1, the Col2, the vinculin (Vcl), the CRABP II, the stathmin (STMN1), and the cofilin-1 (CFL1). In contrast, TAGLN and Col3 are increased during passages [35]. Proteins that showed dysregulated levels along the passages were the 26S protease regulatory subunit 7 (PSMD7), the ubiquitin carboxyl terminal hydrolase isoenzyme L1 (UCH-L1), the heterogeneous nuclear ribonuclear protein H (hnRNP H), and the TAR DNA-binding protein 43 (TDP-43) [35].

In 2007, the proteomic map of human AF-MSCs was constructed and directly compared to the one derived from BM-MSCs [2]. 261 different proteins were identified in AF-MSCs with the majority of the proteins localized in the cytoplasm (41%), whereas others were found in the endoplasmic reticulum (8%), nucleus (13%), mitochondria (12%), ribosomes (1%), cytoskeleton (6%), cytoplasm and the nucleus (5%), and secreted (2%) proteins [2]. AF-MSCs expressed a number of proteins related to proliferation and cell maintenance, such as ubiquilin-1 (UBQLN1), which is known to control cell cycle progression and cell growth, the proliferation associated protein 2G4 (PA2G4), a nucleolar growth-regulating protein, the secreted protein acidic and rich in cysteine (SPARC), which is regulated during embryogenesis and is involved in the control of the cell cycle and cell adhesion, and the enhancer of rudimentary homolog (ERH) that also regulates cell cycle [2]. TAGLN and galectin 1 (Gal 1), both present in stem cells and related to differentiation, were also abundantly expressed in AF-MSCs. Other proteins expressed in high levels in AF-MSCs were related to (i) development, such as Deltex-3-like (DTX3L), and (ii) cytoskeletal organization and movement, such as CFL1, the coactosin-like protein (CLP), and the enabled protein homolog (Enah). As expected, Vim was also expressed in high amounts in AF-MSCs. In this study, a detailed comparison of the common identified proteins in AF cells [58] and AF-MSCs was also described [2].

In our later study [9], we established the proteomic map of the two morphologically distinct AF mesenchymal progenitor cell types (SS and RS) by 2-DE. Twenty-five proteins were differentially expressed in the two subpopulations. Proteins upregulated in SS-AF-MSCs compared to RS-AF-MSCs included reticulocalbin-3 precursor (RCN3), collagen *α*1 (I) (COL1*α*1), FK506-binding protein 9 precursor (FKBP9), Rho GDP-dissociation inhibitor 1 (RhoGDI), chloride intracellular channel protein 4 (CLIC4), tryptophanyl-tRNA synthetase (TrpRS), and 70 kD heat shock protein (HSP70). Peroxiredoxin 2 (Prdx2), 60 kD heat shock protein (HSP60), GSTP, and Anx4 were upregulated in RS-AFMPCs. However, proteins identified in RS-AF-MSCs only included cytokeratin-8, -18, and -19 (CK-8, -18, and CK-19), cathepsin B (CTSB), CLP, and integrin *α*V protein (CD51). Mesenchymal-related proteins, such as Vim, Gal, Gsn, and prohibitin (PHB), were expressed at the same levels in both populations [9].

### 4.2. Amniotic Membrane Stem Cells

A detailed approach for studying human AM proteins was described by Hopkinson et al. [61]. In this study, the authors performed a proteomic analysis of AM samples that were prepared for human transplantation, by using 2-DE gels. The wash media from the AM samples were also examined and the secreted proteins were identified. Proteins detected in both AM and the wash media suggested that partial protein release had occurred. These proteins were mostly soluble cytoplasmic proteins and were categorized according to their subcellular localization and function [61]. One example of the most abundant and consistent proteins in AM is THBS1 which is reported to play role in wound repair, inflammatory response, and angiogenesis [62, 63]. Mimecan (also named osteoglycin/OGN) is another protein detected in AM that represents a small leucine-rich proteoglycan, found in the ECM of connective tissue. Mimecan is reported to maintain the tensile strength and hydration of the tissue [61, 64–66]. In addition, the larger form of mimecan was expressed in AM cells and was susceptible to proteolytic cleavage [65]. TGF-*β*-induced protein ig-h3 (*β*IG-H3), an ECM adhesive molecule acting as a membrane-associated growth factor during cell differentiation and wound healing, and intergrin *α*6 (CD49f), a component of *α*6*β*4 integrin, were also present in significant amounts in AM cells [61, 67, 68]. It is well known that *α*6*β*4-*β*IG-H3 interaction plays an important role in mediating cell adhesion and wound repair signaling pathways [69].

Another important study by Baharvand et al. [70] was focused on the analysis of epithelium-denuded human AM showing both quantitative and qualitative differences compared to nontreated AM [61]. They investigated the proteome of the human AM epithelium, which was used as a limbal stem cell niche for treating ocular surface reconstruction [71, 72]. 515 spots were detected in all the 2-DE gels and 43 proteins were identified using MALDI TOF/TOF MS in AM. The most abundant proteins were different isoforms of lumican (LUM) and OGN, both members of the proteoglycan (PG) family. In particular, OGN might play role in many biological processes including cell growth, angiogenesis, and inflammation [66]. Other proteins detected included collagen VI *α*-1/*α*-2 (Col6a1/Col6a2), fibrinogen beta chain (FGB), transglutaminase 2 isoform A (TGM2A), b-actin variant (ACTB), 70 kD heat shock protein 5 (HSPA5), nidogen 2 (NID2), CD49f, *β*IG-H3, and tubulointerstitial nephritis (TIN) [70]. Some of the proteins identified in this study were also related to extracellular matrix (ECM). Among the detected ones, fibronectin (FN), laminins, and collagen IV (Col4) and VII were reported to promote epithelial adhesion and migration [73, 74].

## 5. Secretome

Recently, significant progress has been made regarding the analysis of the secreted proteins from AFSCs. It has been documented that AFSC secretome was responsible for enhancing vasculogenesis and was capable of evoking a strong angiogenic response in murine recipients [75]. According to this study, a detailed analysis of the AFSC-conditioned media revealed the presence of known proangiogenic and antiangiogenic factors using Luminex's MAP Technology. Vascular endothelial growth factor (VEGF), stromal cell-derived factor 1 (SDF-1), interleukin 8 (IL-8), monocyte chemotactic protein 1 (MCP-1), and two angiogenesis inhibitors, interferon-gamma (IFN*γ*) and interferon gamma-induced protein 10 (IP-10), were identified as secreted proteins [75–77]. It was also demonstrated that a relative small number of AFSC was enough to secrete a detectable amount of proangiogenic growth factors and cytokines. The secretion of these can be regulated in a dose-dependent manner according to the initial cell number of the cells used [24, 75].

A systematic study on AFSC-secreted proteins led to the conclusion that proangiogenic soluble factors from AFSCs can mediate the recruitment of endothelial progenitors in an ischemic rat model [78]. In particular, conditioned medium derived from AFSCs could topically deliver angiogenic growth factors and cytokines into the skin flap of the ischemic rat model and was responsible for triggering the endogenous repair by recruiting endothelial progenitor cells [78].

In our recent studies, we examined the therapeutic potential of an AF-MSCs and their secreted molecules in mice with acute hepatic failure [24]. A variety of cytokines and growth factor were detected in AF-MSC conditioned medium. Cytokines such as interleukin 10 (IL-10), interleukin 27 (IL-27), interleukin 17 family (IL-17E), interleukin 12p70 (IL-12p70), interleukin-1 beta (IL-1*β*), and interleukin-1 receptor antagonist (IL-1ra), responsible for inducing local and systemic downregulation of pro-inflammatory mediators, were detected. SERPINE1, MCP-1, and SDF-1, responsible for promoting tissue repair, were also secreted [24, 79, 80]. Interestingly, among the highly expressed growth factors were platelet-derived endothelial cell growth factor (PD-ECGF), endostatin/collagen XVII (EN/Col17), urinary plasminogen activator (uPA), TIMP1, TIMP2, heparin-binding EGF-like growth factor (HB-EGF), fibroblast growth factor 7 (FGF7), and epidermal growth factor (EGF), responsible for liver regeneration and tissue repair [24, 81].

## 6. Summary

**Figure 1:**
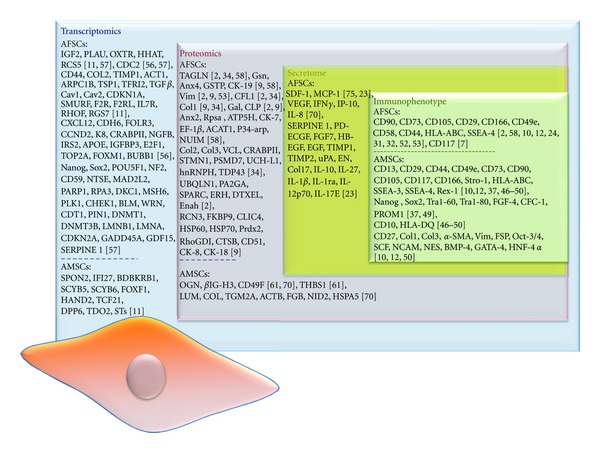
Summary of the most important markers identified in AFCs and AMCs by the use of transcriptomics, proteomics, secretome, and immunophenotypic analyses. Proteins identified in more than one study are marked in bold.

The current data so far suggest that amniotic fluid and amniotic membrane may represent promising sources for stem cells of mesenchymal origin. Indeed, MSCs are more abundant and a wide range of protocols has been described for their isolation. However, it is reported that different culture conditions of the same type of cells may affect their differential gene expression pattern, which represents a limitation for their isolation and expansion in vitro. Studies including phenotypic analysis, using methodologies such as flow cytometry and immunohistochemistry, as well as transcriptomics, proteomics, and secretome analyses approaches, aim to determine the protein profile of these cells (Figure 1). Data generated by such studies are expected to clarify their differential repertoire and validate the molecular profile of these stem cells. However, the main issue urged to be addressed is the isolation of a homogenous population that may facilitate systematic studies for the elucidation of the function of these multipotent cells.

Such approaches may lead to the identification of key antigens that mirror the phenotype of these cells and explain their distinct features properties. This type of studies will open the way for a systematic and efficient isolation of these cells prior to their use at the clinical setting.

## Appendix

### Questions for Further Investigation

Which are the appropriate isolation methods and culture conditions of AFSCs or AMSCs that will allow the identification of a consistent phenotype?

Is there a single marker that can be used for AFSCs or AMSCs isolation?

The AFSC and AMSC populations are heterogeneous and differ in their phenotypic and molecular properties. Methods of isolation can result in a homogeneous cell population.

AFSCs or AMSCs can be used as tools in regenerative medicine: establishment of culture conditions with minimal or no animal substances.

Marker Discovery The AFSCs and the AMSCs initial characterization can be performed by immunophenotype analysis by using well-characterized cell surface markers such as AFSCs: CD90, CD73, CD105, CD29, CD166, CD49e, CD58, CD44, HLA-ABC, SSEA-4; AMSCs: CD13, CD29, CD44, CD49e, CD73, CD90, CD105, CD117, CD166, Stro-1, HLA-ABC, SSEA-3, SSEA-4, Nanog, Sox2, Tra1-60, Tra1-80, FGF-4, CFC-1, and PROM1.

Transcriptomics and Proteomics Revealed the Identification of Key Markers Expressed such as AFSCs: Nanog, Sox2, POU5F1, NF2, IGF2, PLAU, OXTR, HHAT, RCS5, CDC2, COL2, TAGLN, Gsn, Anx4, GSTP, CK-19, Vim, Col1, and Gal; AMSCs: OGN, *β*IG-H3, and CD49F. Since there is no common marker available for AFSC and AMSC, a wider panel of markers needs to be employed. This also urges the conduction of further detailed array and functional analyses in order to define the most appropriate markers for AFSC and AMSC characterization.

## References

[1] Fauza D (2004). Amniotic fluid and placental stem cells. *Best Practice and Research*.

[2] Roubelakis MG, Pappa KI, Bitsika V (2007). Molecular and proteomic characterization of human mesenchymal stem cells derived from amniotic fluid: comparison to bone marrow mesenchymal stem cells. *Stem Cells and Development*.

[3] Klemmt PA, Vafaizadeh V, Groner B (2011). The potential of amniotic fluid stem cells for cellular therapy and tissue engineering. *Expert Opinion on Biological Therapy*.

[4] In’t Anker PS, Scherjon SA, Kleijburg-van der Keur C (2003). Amniotic fluid as a novel source of mesenchymal stem cells for therapeutic transplantation [1]. *Blood*.

[5] Tsai MS, Lee JL, Chang YJ, Hwang SM (2004). Isolation of human multipotent mesenchymal stem cells from second-trimester amniotic fluid using a novel two-stage culture protocol. *Human Reproduction*.

[6] Tsai MS, Hwang SM, Tsai YL, Cheng FC, Lee JL, Chang YJ (2006). Clonal amniotic fluid-derived stem cells express characteristics of both mesenchymal and neural stem cells. *Biology of Reproduction*.

[7] De Coppi P, Bartsch G, Siddiqui MM (2007). Isolation of amniotic stem cell lines with potential for therapy. *Nature Biotechnology*.

[8] Prusa AR, Hengstschläger M (2002). Amniotic fluid cells and human stem cell research - A new connection. *Medical Science Monitor*.

[9] Roubelakis MG, Bitsika V, Zagoura D (2011). In vitro and in vivo properties of distinct populations of amniotic fluid mesenchymal progenitor cells. *Journal of Cellular and Molecular Medicine*.

[10] Kim J, Kang HM, Kim H (2007). Ex vivo characteristics of human amniotic membrane-derived stem cells. *Cloning and Stem Cells*.

[11] Tsai MS, Hwang SM, Chen KD (2007). Functional network analysis of the transcriptomes of mesenchymal stem cells derived from amniotic fluid, amniotic membrane, cord blood, and bone marrow. *Stem Cells*.

[12] Pappa KI, Anagnou NP (2009). Novel sources of fetal stem cells: where do they fit on the developmental continuum?. *Regenerative Medicine*.

[13] Bollini S, Cheung KK, Riegler J (2011). Amniotic fluid stem cells are cardioprotective following acute myocardial infarction. *Stem Cells and Development*.

[14] Hauser PV, De Fazio R, Bruno S (2010). Stem cells derived from human amniotic fluid contribute to acute kidney injury recovery. *American Journal of Pathology*.

[15] Lee WY, Wei HJ, Lin WW (2011). Enhancement of cell retention and functional benefits in myocardial infarction using human amniotic-fluid stem-cell bodies enriched with endogenous ECM. *Biomaterials*.

[16] Maraldi T, Riccio M, Resca E, Pisciotta A, La Sala GB (2011). Human amniotic fluid stem cells seeded in fibroin scaffold produce in vivo mineralized matrix. *Tissue Engineering Part A*.

[17] Shaw SWS, David AL, De Coppi P (2011). Clinical applications of prenatal and postnatal therapy using stem cells retrieved from amniotic fluid. *Current Opinion in Obstetrics and Gynecology*.

[18] Turner CG, Klein JD, Steigman SA (2011). Preclinical regulatory validation of an engineered diaphragmatic tendon made with amniotic mesenchymal stem cells. *Journal of Pediatric Surgery*.

[19] Angelini A, Castellani C, Ravara B (2011). Stem-cell therapy in an experimental model of pulmonary hypertension and right heart failure: role of paracrine and neurohormonal milieu in the remodeling process. *Journal of Heart and Lung Transplantation*.

[20] Bitsika V, Roubelakis MG, Zagoura D, Trohatou O, Makridakis M Human amniotic fluid-derived mesenchymal stem cells as therapeutic
vehicles: a novel approach for the treatment of bladder cancer.

[21] Perin L, Sedrakyan S, Giuliani S (2010). Protective effect of human amniotic fluid stem cells in an immunodeficient mouse model of acute tubular necrosis. *PLoS ONE*.

[22] Rosner M, Schipany K, Gundacker C, Shanmugasundaram B, Li K Renal differentiation of amniotic fluid stem cells: perspectives for clinical application and for studies on specific human genetic diseases.

[23] Rota C, Imberti B, Pozzobon M, Piccoli M, De Coppi P human amniotic fluid stem cell preconditioning improves their
regenerative potential.

[24] Zagoura DS, Roubelakis MG, Bitsika V, Trohatou O, Pappa KI Therapeutic potential of a distinct population of human amniotic fluid mesenchymal stem cells and their secreted molecules in mice with acute hepatic failure.

[25] Gosden CM (1983). Amniotic fluid cell types and culture. *British Medical Bulletin*.

[26] Hoehn H, Salk D (1982). Morphological and biochemical heterogeneity of amniotic fluid cells in culture. *Methods in Cell Biology*.

[27] Manuelpillai U, Moodley Y, Borlongan CV, Parolini O (2011). Amniotic membrane and amniotic cells: potential therapeutic tools to combat tissue inflammation and fibrosis?. *Placenta*.

[28] Leschot NJ, Verjaal M, Treffers PE (1985). Risks of midtrimester amniocentesis; assessment in 3000 pregnancies. *British Journal of Obstetrics and Gynaecology*.

[29] Eddleman KA, Malone FD, Sullivan L (2006). Pregnancy loss rates after midtrimester amniocentesis. *Obstetrics and Gynecology*.

[30] In’t Anker PS, Scherjon SA, Kleijburg-Van Der Keur C (2004). Isolation of mesenchymal stem cells of fetal or maternal origin from human placenta. *Stem Cells*.

[31] Cipriani S, Bonini D, Marchina E (2007). Mesenchymal cells from human amniotic fluid survive and migrate after transplantation into adult rat brain. *Cell Biology International*.

[32] Kim J, Lee Y, Kim H (2007). Human amniotic fluid-derived stem cells have characteristics of multipotent stem cells. *Cell Proliferation*.

[33] Bossolasco P, Montemurro T, Cova L (2006). Molecular and phenotypic characterization of human amniotic fluid cells and their differentiation potential. *Cell Research*.

[34] Ditadi A, De Coppi P, Picone O (2009). Human and murine amniotic fluid c-Kit+Lin- cells display hematopoietic activity. *Blood*.

[35] Chen WQ, Siegel N, Li L, Pollak A, Hengstschläger M, Lubec G (2009). Variations of protein levels in human amniotic fluid stem cells CD117/2 over passages 5-25. *Journal of Proteome Research*.

[36] Sessarego N, Parodi A, Podestà M (2008). Multipotent mesenchymal stromal cells from amniotic fluid: solid perspectives for clinical application. *Haematologica*.

[37] Delo DM, De Coppi P, Bartsch G, Atala A (2006). Amniotic fluid and placental stem cells. *Methods in Enzymology*.

[38] Ilancheran S, Michalska A, Peh G, Wallace EM, Pera M, Manuelpillai U (2007). Stem cells derived from human fetal membranes display multilineage differentiation potential. *Biology of Reproduction*.

[39] Bose B (1979). Burn wound dressing with human amniotic membrane. *Annals of the Royal College of Surgeons of England*.

[40] Hao Y, Ma DHK, Hwang DG, Kim WS, Zhang F (2000). Identification of antiangiogenic and antiinflammatory proteins in human amniotic membrane. *Cornea*.

[41] Arai N, Tsuno H, Okabe M, Yoshida T, Koike C clinical application of a hyperdry amniotic membrane on surgical
defects of the oral mucosa.

[42] Kim JC, Tseng SCG (1995). Transplantation of preserved human amniotic membrane for surface reconstruction in severely damaged rabbit corneas. *Cornea*.

[43] Kitagawa K, Okabe M, Yanagisawa S, Zhang XY, Nikaido T, Hayashi A (2011). Use of a hyperdried cross-linked amniotic membrane as initial therapy for corneal perforations. *Japanese Journal of Ophthalmology*.

[44] Kitagawa K, Yanagisawa S, Watanabe K (2009). A hyperdry amniotic membrane patch using a tissue adhesive for corneal perforations and bleb leaks. *American Journal of Ophthalmology*.

[45] Iijima K, Igawa Y, Imamura T (2007). Transplantation of preserved human amniotic membrane for bladder augmentation in rats. *Tissue Engineering*.

[46] Cai J, Li W, Su H (2010). Generation of human induced pluripotent stem cells from umbilical cord matrix and amniotic membrane mesenchymal cells. *Journal of Biological Chemistry*.

[47] Parolini O, Alviano F, Bagnara GP (2008). Concise review: isolation and characterization of cells from human term placenta: outcome of the First International Workshop on Placenta Derived Stem Cells. *Stem Cells*.

[48] Miki T, Marongiu F, Dorko K, Ellis ECS, Strom SC (2010). Isolation of amniotic epithelial stem cells. *Current Protocols in Stem Cell Biology*.

[49] Soncini M, Vertua E, Gibelli L (2007). Isolation and characterization of mesenchymal cells from human fetal membranes. *Journal of Tissue Engineering and Regenerative Medicine*.

[50] Marongiu F, Gramignoli R, Sun Q (2010). Isolation of amniotic mesenchymal stem cells. *Current Protocols in Stem Cell Biology*.

[51] Miki T, Mitamura K, Ross MA, Stolz DB, Strom SC (2007). Identification of stem cell marker-positive cells by immunofluorescence in term human amnion. *Journal of Reproductive Immunology*.

[52] Prusa AR, Marton E, Rosner M, Bernaschek G, Hengstschläger M (2003). Oct-4-expressing cells in human amniotic fluid: a new source for stem cell research?. *Human Reproduction*.

[53] Zhao P, Ise H, Hongo M, Ota M, Konishi I, Nikaido T (2005). Human amniotic mesenchymal cells have some characteristics of cardiomyocytes. *Transplantation*.

[54] Toda A, Okabe M, Yoshida T, Nikaido T (2007). The potential of amniotic membrane/amnion-derived cells for regeneration of various tissues. *Journal of Pharmacological Sciences*.

[55] Takashima S, Yasuo M, Sanzen N (2008). Characterization of laminin isoforms in human amnion. *Tissue and Cell*.

[56] Kim YW, Kim HJ, Bae SM, Kim YJ, Shin JC (2010). Time-course transcriptional profiling of human amniotic fluid-derived stem cells using microarray. *Cancer Research and Treatment*.

[57] Wolfrum K, Wang Y, Prigione A, Sperling K, Lehrach H, Adjaye J (2010). The LARGE principle of cellular reprogramming: lost, acquired and retained gene expression in foreskin and amniotic fluid-derived human iPS cells. *PLoS ONE*.

[58] Tsangaris G, Weitzdörfer R, Pollak D, Lubec G, Fountoulakis M (2005). The amniotic fluid cell proteome. *Electrophoresis*.

[59] Oh JE, Fountoulakis M, Juranville JF, Rosner M, Hengstschlaeger M, Lubec G (2004). Proteomic determination of metabolic enzymes of the amnion cell: basis for a possible diagnostic tool?. *Proteomics*.

[60] Kim SH, Vlkolinsky R, Cairns N, Lubec G (2000). Decreased levels of complex III core protein 1 and complex V *β* chain in brains from patients with Alzheimer’s disease and down syndrome. *Cellular and Molecular Life Sciences*.

[61] Hopkinson A, McIntosh RS, Shanmuganathan V, Tighe PJ, Dua HS (2006). Proteomic analysis of amniotic membrane prepared for human transplantation: characterization of proteins and clinical implications. *Journal of Proteome Research*.

[62] Adams JC, Lawler J (2004). The thrombospondins. *International Journal of Biochemistry and Cell Biology*.

[63] Zaslavsky A, Baek KH, Lynch RC (2010). Platelet-derived thrombospondin-1 is a critical negative regulator and potential biomarker of angiogenesis. *Blood*.

[64] Funderburgh JL, Corpuz LM, Roth MR, Funderburgh ML, Tasheva ES, Conrad GW (1997). Mimecan, the 25-kDa corneal keratan sulfate proteoglycan, is a product of the gene producing osteoglycin. *Journal of Biological Chemistry*.

[65] Tasheva ES, Koester A, Paulsen AQ (2002). Mimecan/osteoglycin-deficient mice have collagen fibril abnormalities. *Molecular Vision*.

[66] Kampmann A, Fernández B, Deindl E (2009). The proteoglycan osteoglycin/mimecan is correlated with arteriogenesis. *Molecular and Cellular Biochemistry*.

[67] Kim MO, Yun SJ, Kim IS, Sohn S, Lee EH (2003). Transforming growth factor-*β*-inducible gene-h3 (*β*ig-h3) promotes cell adhesion of human astrocytoma cells in vitro: implication of *α*6*β*4 integrin. *Neuroscience Letters*.

[68] Corsini NS, Martin-Villalba A (2010). Integrin alpha 6: anchors away for glioma stem cells. *Cell Stem Cell*.

[69] Endo KI, Nakamura T, Kawasaki S, Kinoshita S (2004). Human amniotic membrane, like corneal epithelial basement membrane, manifests the *α*5 chain of type IV collagen. *Investigative Ophthalmology and Visual Science*.

[70] Baharvand H, Heidari M, Ebrahimi M, Valadbeigi T, Salekdeh GH (2007). Proteomic analysis of epithelium-denuded human amniotic membrane as a limbal stem cell niche. *Molecular Vision*.

[71] Grueterich M, Espana EM, Tseng SCG (2003). Ex vivo expansion of limbal epithelial stem cells: amniotic membrane serving as a stem cell niche. *Survey of Ophthalmology*.

[72] Li W, He H, Kuo CL, Gao Y, Kawakita T, Tseng SCG (2006). Basement membrane dissolution and reassembly by limbal corneal epithelial cells expanded on amniotic membrane. *Investigative Ophthalmology and Visual Science*.

[73] Fukuda K, Chikama TI, Nakamura M, Nishida T (1999). Differential distribution of subchains of the basement membrane components type IV collagen and laminin among the amniotic membrane, cornea, and conjunctiva. *Cornea*.

[74] Lobert VH, Brech A, Pedersen NM (2010). Ubiquitination of *α*5*β*1 integrin controls fibroblast migration through lysosomal degradation of fibronectin-integrin complexes. *Developmental Cell*.

[75] Teodelinda M, Michele C, Sebastiano C, Ranieri C, Chiara G (2011). Amniotic liquid derived stem cells as reservoir of secreted angiogenic factors capable of stimulating neo-arteriogenesis in an ischemic model. *Biomaterials*.

[76] Charo IF, Taubman MB (2004). Chemokines in the pathogenesis of vascular disease. *Circulation Research*.

[77] Heil M, Schaper W (2005). Arteriogenic growth factors, chemokines and proteases as a prerequisite for arteriogenesis. *Drug News and Perspectives*.

[78] Mirabella T, Hartinger J, Lorandi C, Gentili C, van Griensven M Pro-angiogenic soluble factors from amniotic fluid stem cells mediate the recruitment of endothelial progenitors in a model of ischemic fasciocutaneous flap.

[79] Maroof A, Kaye PM (2008). Temporal regulation of interleukin-12p70 (IL-12p70) and IL-12-related cytokines in splenic dendritic cell subsets during Leishmania donovani infection. *Infection and Immunity*.

[80] Yoshidome H, Kato A, Miyazaki M, Edwards MJ, Lentsch AB (1999). IL-13 activates STAT6 and inhibits liver injury induced by ischemia/reperfusion. *American Journal of Pathology*.

[81] van Poll D, Parekkadan B, Borel Rinkes IHM, Tilles AW, Yarmush ML (2008). Mesenchymal stem cell therapy for protection and repair of injured vital organs. *Cell and Molecular Biology*.

